# Reassessment of Porosimetry Determinations Using Water Vapor Sorption Measurements for Pastes and Concretes Containing Basaltic Aggregates Compared to the Mercury Intrusion Method

**DOI:** 10.3390/ma18102257

**Published:** 2025-05-13

**Authors:** Natalia Rodríguez-Brito, Concepción Blanco-Peñalver, Ricardo M. Souto, Carmen Andrade, Juan J. Santana

**Affiliations:** 1 Consejería de Obras Públicas y Transportes, Gobierno de Canarias, 38270 La Laguna, Spain; nrodbrib@gobiernodecanarias.org (N.R.-B.); cblapen@gobiernodecanarias.org (C.B.-P.); 2 Department of Chemistry, Universidad de La Laguna, P.O. Box 456, 38200 La Laguna, Spain; rsouto@ull.edu.es; 3 Institute of Material Science and Nanotechnology, Universidad de La Laguna, 38200 La Laguna, Spain; 4 CIMNE—International Center for Numerical Methods in Engineering, 28010 Madrid, Spain; 5 Department of Process Engineering, University of Las Palmas de Gran Canaria, 35017 Las Palmas de Gran Canaria, Spain

**Keywords:** cement paste, concrete, mercury intrusion porosity, water-accessible porosity, compressive strength, durability

## Abstract

Concrete porosity is one of the fundamental properties for the structural characterization of cementitious materials. This study compares porosity data obtained with dynamic water vapor sorption (DWVS) with the more commonly used mercury intrusion porosimetry (MIP) method for a wide range of concrete samples made with basaltic aggregates, typical of the Canary Islands, which are porous. The objective was to propose an alternative method for routine concrete monitoring that avoids the use of a hazardous substance such as mercury. The results reveal fundamental differences between the MIP and water-accessible porosimetry (WAP) data, although a correlation between the methods was revealed where MIP = 1.18 × WAP. The study was completed by an analysis of the relationships between the porosity and the characteristics and properties of concrete (water/cement ratio and strength), as well as the calculation of the tortuosity factor and a frost durability factor.

## 1. Introduction

The hydrated cement used to make concrete is a relatively highly porous material, with a pore size distribution ranging from nanometers to micrometers [1]. The pores have an irregular geometry, and their number and the connectivity between them determine some material properties, such as the compressive strength, and influence its durability [2], making them permeable materials [3]. However, with a relatively low water/cement (*w*/*c*) ratio and a high degree of hydration of the paste, the pores remain no longer interconnected, and the permeability of the concrete decreases drastically compared to that obtained at early age [4,5,6,7].

Porosity is not a static value [8,9], it evolves with the degree of hydration and also depends on the interaction of the material with the environment [10]. Thus, for example, carbonation or the ingress of chloride can alter the porosity by a reaction with existing cement hydrates, leading to the formation of new phases that precipitate in the pores [11,12,13]. Therefore, if the porosity is to be compared or used to characterize concrete or for any subsequent calculations [14,15], it must be associated with the concrete age and the specific service conditions [9,16]. On the other hand, the measurement technique employed can also alter the porosity, either during the preconditioning of the sample [17] (e.g., the necessary drying [18,19]) or by the operation of the technique itself, as is the case with the high pressures required for mercury intrusion [20,21,22,23], or the swelling caused by solvent displacement drying [24].

The most common method for characterizing porosity is currently mercury intrusion porosimetry (MIP) [25,26,27,28,29,30], as it allows the characterization of various aspects of the pore structure, such as the pore size distribution, specific surface, and bulk density. This technique allows the measurement of a wide range of pore sizes (typically 0.003–375 µm) [18] in the capillary pore range, but there is controversy as to whether [31,32] or not [23,24,30,33] it provides an accurate pore size distribution at all scales. Among the reasons cited for limiting the applicability of the method, the most important are the following:

- The effect of high pressures that can damage pores during the intrusion process [20,21,22,23];
- The so-called ink-bottle effect [9,31], which prevents the determination of some larger pores when they are accessible only through smaller pores [31];
- The assumed contact angle of mercury with the cement-based material [27,31,34];
- The requisite that samples be dried, which may change the pore structure during preconditioning [21,35].

The aim of this paper is not to perform an exhaustive analysis or to study the limitations of the MIP technique, because, despite these limitations, it is widely used in studies of the porous microstructure of concrete. Indeed, the threshold pore size provides valuable information on the permeability and diffusivity of a cement paste [36], and under certain pressure conditions that do not cause the smallest pores to collapse or break when reaching isolated pores, the porosity value can very well represent the real microstructure of the concrete [20,37,38,39,40].

However, mercury is a hazardous substance for human health, and its use should be avoided and therefore replaced by another substance for the characterization of concrete. In order to replace MIP and find a much more economical and easy-to-use method, the water-accessible porosity technique was selected among other existing techniques [41,42]. The dynamic water vapor sorption (DWVS) technique operates under vacuum conditions using water as the intrusive liquid. Although water saturation by capillary absorption can also be used as an alternative, it was discarded in this work because it is much slower. Indeed, complete saturation of the sample can take weeks and be impaired by swelling [43,44]. In contrast, the technique based on saturation under vacuum is very accessible and has the advantage of being reversible. Therefore, the samples can be used for other tests. However, its main limitation is that only provides the total porosity, without giving information on the size distribution and internal geometry of the pores.

Although the DWVS method has been recommended as an alternative to MIP for the microstructural characterization of cementitious materials [45], few works have compared the data obtained with these two techniques [46,47,48]. When comparing three ASTM methods of measuring the porosity using water in [46], only two concrete samples were used. Among cold water saturation, boiling water saturation, and vacuum saturation, the authors presented vacuum saturation as the most effective. Then, in [47], the porosity by MIP and by water vacuum saturation were compared, finding that the latter presented higher porosity values. But in [48], the opposite result was obtained, where the porosity using water was lower than that obtained by MIP, probably because in this work, vacuum saturation was not used, but rather conditioning at 100% relative humidity until equilibrium was reached.

This lack of systematic data motivated this work, which focuses on comparing the total porosity obtained by both techniques with a view to avoiding the use of hazardous substances and on the simplicity of the DWVS method compared to MIP, which would facilitate its implementation in quality control laboratories. The comparison was carried out with concrete samples as well as their pastes, since the presence of aggregates can introduce differences in the proportion of paste in MIP samples, which are much smaller in size than those of water-accessible porosimetry (WAP). Based on these results, complementary analyses were carried out to study the relationship between porosity values and strength. In addition, the tortuosity factor of the different concretes was characterized from the MIP results, using an equation proposed in a previous publication [49].

## 2. Materials and Methods

**Figure 1:**
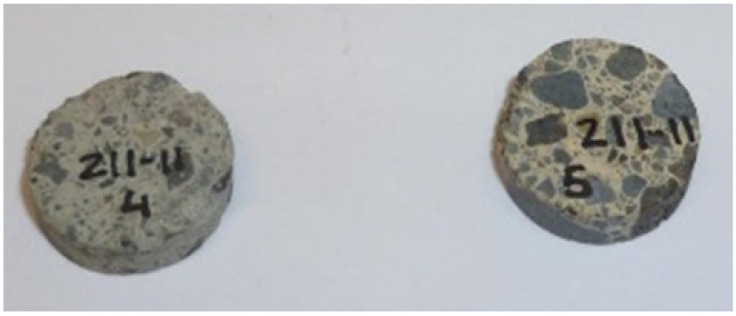
Two concrete samples used for WAP measurements.

All cement paste measurements were performed on duplicate samples, and on triplicate samples for concrete samples, to validate the reliability of the results. In all cases, these analyses were performed on samples from the same batch. Surface tension and contact angle values were taken from the literature. Figure 1 shows two concrete samples prepared for MIP measurements.

### 2.1. Materials

As shown in Table 1, six pastes of different types of cement, coded from C1 to C6, thirty-seven concretes of different compressive strength and characteristics, coded from H1 to H36, and one sample of typical basaltic aggregates (called A1) were analyzed. The aggregates in all concrete samples were the characteristic basalt from Tenerife (Canary Islands, Spain). These concretes were prepared on site in industrial concrete plants and are therefore not “lab-crete” samples. They were taken from large batches, under real fabrication conditions, in large mixers of different sizes, and cured in accordance with regulations [50,51]. Table 2 shows the material proportions for the concrete samples. Concrete composition ranges from a cement content of 240 to 250 kg/m^3^, with a water/binder ratio of 0.63 and a strength of 12.18 MPa, to a cement content of 500 kg/m^3^, with a water/binder ratio of 0.28 and a strength of 58.55 MPa. There is therefore a wide variety of concrete types.

The pastes and concretes were kept in the mold for 24 h in a humid chamber with a relative humidity greater than 95% and a temperature of 20 ± 2 °C. After demolding, they remained in the humid chamber for up to 28 days, before being conditioned for the MIP test or dried for the water-accessible porosity test.

### 2.2. Mercury Intrusion Technique (MIP)

The tests were carried out in accordance with the specifications of ASTM D4404 [52], using 1 cm^3^ cylindrical samples taken from specimens used in other tests that have not undergone deterioration (Figure 1 shows photographs of two typical samples). The samples were dried in an oven at 100 °C for 48 h before testing and then placed in a vacuum chamber. Hydrostatic pressure was then applied, thus increasing the intrusion of mercury.

A PORESIZER Autopore IV 9500 (Micromeritics, Norcross, GA, USA) porosimeter was used. During testing, the applied pressure ranged from 3.6 × 10^−3^ to 4.098 × 10^8^ MPa, allowing the characterization of pores from 3.6 nm to 412 µm. The applied surface tension and contact angle values were 0.484 N/m and 141°, respectively, very close to those reported in the literature.

### 2.3. Porosity Accessible by Water

Porosity quantification by the water-accessibility test is a standardized technique [41,42] used to quantify the open porosity of natural stones and is also defined as the water-accessible porosity (WAP) for concrete. The procedure consists of drying a sample of cement paste or concrete to a constant mass (*m_d_*) at a temperature of 100 °C, then placing it in a vacuum container, where the pressure is gradually decreased to 2.0 ± 0.7 kPa, and maintained for 2 h to remove the air contained in the open pores of the samples. Under these same vacuum conditions, demineralized water at 20 °C is slowly introduced into the container (15 min). When all the samples are submerged, atmospheric pressure is restored in the container, and they are maintained under these conditions for another 24 h. The samples are then removed from the container, and excess water is quickly removed from the surface with a damp cloth, and the samples are weighed, obtaining the saturated mass (*m_s_*). Finally, the samples are weighed by placing them on a hydrostatic balance to measure the mass of the specimen submerged under water (*m_h_*).

The tested samples come from prismatic specimens of 4 × 4 × 16 cm^3^, cured for 28 days, cut to the appropriate dimensions, and tested within approximately 1 month. Tests carried out according to UNE-EN 1936 [41] require six specimens with a volume of 4 × 4 × 4 cm^3^, while tests carried out according to UNE 83980-14 [42] use two specimens with a weight greater than 800 g and dimensions of 5 × 10 × 10 cm^3^.

## 3. Results

The total porosity data were obtained using the MIP and WAP methods for all the samples under study. Since the procedure employed for porosity quantification is different for each method, possible differences should be identified in order to derive an equivalence between the information supplied in each case. Thus, mercury intrusion porosimetry (MIP) is based on the principle that a non-wetting liquid (with a contact angle greater than 90°), such as mercury, only penetrates capillaries under pressure (several hundred MPa). The relationship between pressure and the capillary diameter (similar to a cylinder) is described by the Washburn–Laplace equation [53]:

(1) $$P=\frac{-4\cdot \gamma \cdot \cos\theta }{d}$$

where *P* = pressure (Pa), *γ* = surface tension of the liquid (N/m), *θ* = contact angle between the liquid and the solid (degrees), and *d* = capillary diameter (m). The pore size distribution is determined from the volume of mercury introduced at each increase in pressure. The total porosity is determined from the total volume introduced.

On the other hand, the procedure used in the WAP technique consists of replacing the air contained in the open pores of a sample with water. This procedure involves initially drying the sample to obtain its dry mass (*m_d_*), followed by removing the air contained in the pores under vacuum and filling them with demineralized water, resulting in the saturated mass (*m_s_*). Finally, the samples are also weighed on a hydrostatic balance to measure the mass of the specimen submerged under water (*m_h_*). Open porosity is then calculated using the expression

(2) $$P_{0}=\frac{m_{s}-m_{d}}{m_{s}-m_{h}}\cdot 100$$

**Figure 2:**
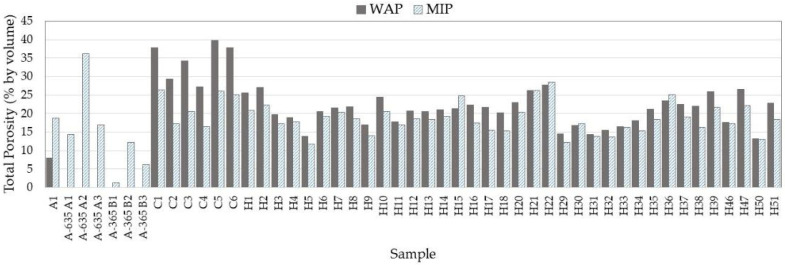
Porosity data obtained using WAP and MIP techniques.

Figure 2 and Table 3 present the total porosity values obtained by the MIP and WAP methods, as well as the compressive strength at 28 days and the dry density of the samples. These data indicate that the water-accessible porosity is generally, but not always, higher than that obtained by MIP. Furthermore, the cement paste samples have higher total porosities than those obtained for concrete, since in the latter, the samples always contain aggregates (although without aggregates larger than 5 mm given the small volume of the samples used for the MIP test). Thus, the porosity range observed for the cement paste samples varies between 27.30% (C4) and 39.83% (C5), while the porosity of the concrete samples varies between 11.68%. (H5) and 28.58% (H22). Only samples H5, H9, H29, H31, H32, and H50 show intermediate porosity (between 10% and 15%).

**Figure 3:**
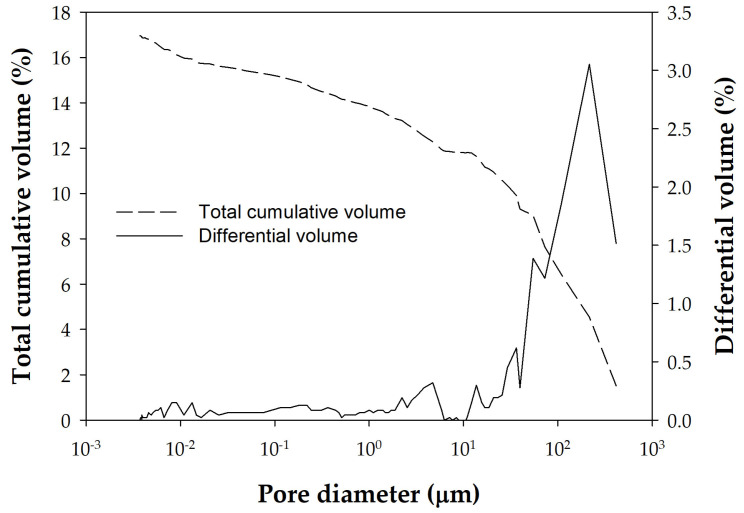
Porosity distribution of the basaltic aggregate from MIP measurements.

Regarding the aggregates, it should be noted in Table 3 and Figure 2 that the aggregate used, A1, of basaltic nature, shows an inverse behavior, its MIP being much higher than that measured by water intrusion. Six other MIP measurements were carried out with different aggregates, and Table 2 and Figure 1 illustrate the variability of the basalts used as aggregates in the Canary Islands. This is reflected in the high variability of the mercury porosimetry data, with some aggregates having very low porosity (1.3%) and others very high porosity (36.2%). Figure 3 shows the MIP of the basaltic aggregate with the lowest porosity (1.3%).

**Figure 4:**
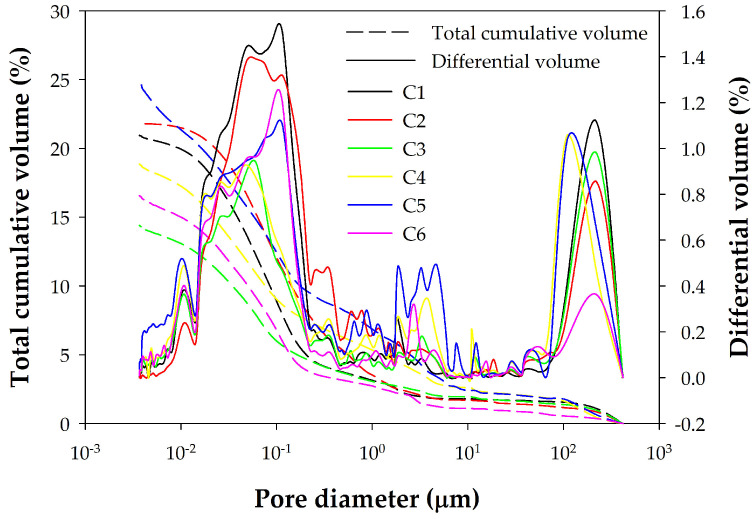
Porosity distribution plots of the investigated pastes from MIP measurements.

**Figure 5:**
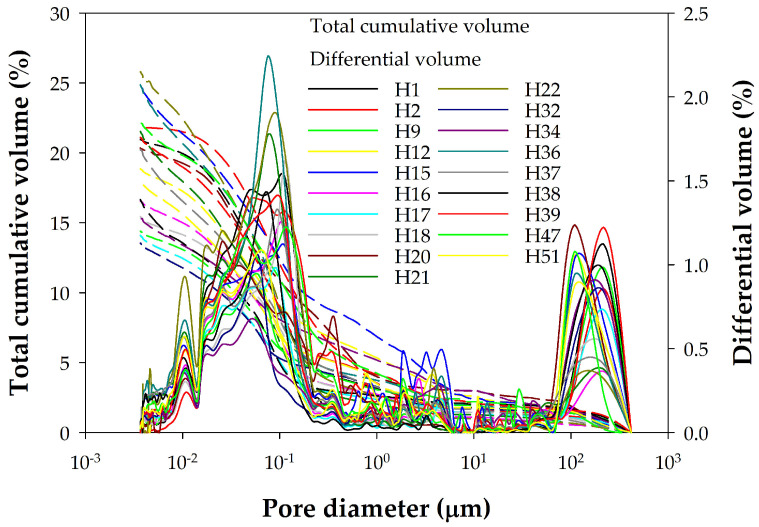
Porosity distribution plots of the investigated concretes from MIP measurements.

**Figure 6:**
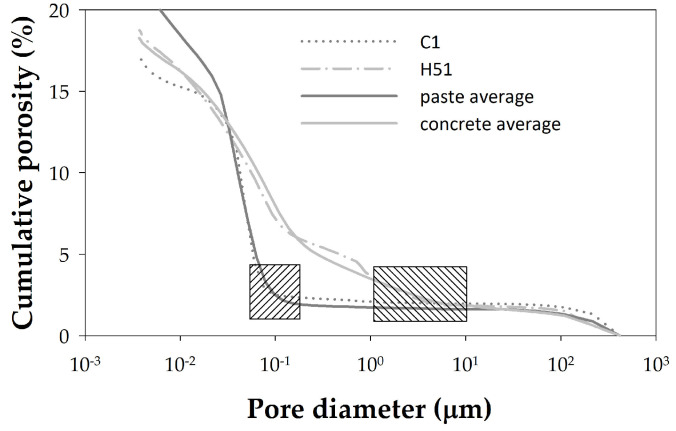
Comparison of the cumulative porosity distributions for samples C1 and H51, as well as for the average values of paste and concrete samples. Data obtained using MIP with *γ* = 0.481 N/m and *θ* = 141°.

Similarly, Figure 4 shows the MIP porosities of the pastes, and Figure 5 shows those of the concretes. Comparing these figures reveals a significant difference between the porosity distributions obtained by MIP for pastes and concretes. Figure 6 allows us to observe this difference in more detail for two selected samples by highlighting with a shaded square the pore sizes for which there are large differences between the distributions of pastes and concretes. The cement paste samples thus present a pore size distribution within a single family centered around pores of size 0.05 µm (Figure 4). Pores larger than 0.1 µm contribute little to the total porosity of the sample, which is concentrated for small diameter values. The final cumulative porosity value shown in the graph corresponds to the total porosity of the sample. In contrast, the concrete samples present a different trend. The porosity is mainly determined by pore diameter values starting from 0.1 µm (Figure 5). Figure 6 shows more clearly that, while pastes have a pore size of less than 0.1 µm, concretes have a pore size threshold around 10 µm, representing almost 40% of the total accumulated porosity for the diameter range from the largest of them to 0.1 µm. This indicates that concrete samples have a broader pore structure, in terms of pore size distribution, compared to cement pastes. This difference seems logically attributable to the paste/aggregate interfaces and to the aggregate itself, because its pores are larger despite its lack of porosity.

## 4. Discussion

Firstly, the relationship between the two methods of porosity measurement will be discussed, and then some relationships between the measured porosity and certain characteristics or properties of concrete will be derived.

### 4.1. Comparative Analysis of MIP and WAP Data

**Figure 7:**
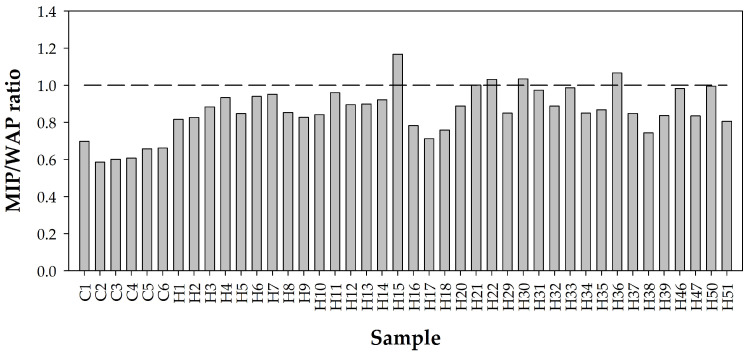
Ratios of the MIP total porosity with respect to that determined with WAP. A value greater than 1 means that MIP is greater than WAP.

Figure 7 shows the ratio of MIP to WAP values, suggesting that WAP gives higher values than MIP, and that cement pastes generally show higher porosity values than concretes. The differences are somewhat random, with the MIP/WAP ratios being significantly lower in the case of pastes, indicating that the aggregate contributes to the porosity of concrete, although in varying proportions. The ratios found between the two porosities range between 0.71 and 1.16, the average being 0.86 with a coefficient of variation of 15%. This is interpreted as the influence of the variability of the aggregate being 10 to 15%, which is considered an acceptable difference and within the accuracies of each measurement method. Since aggregates are generally more impermeable and less variable in their porosity, it is also possible to infer that any other less variable aggregate will give a lower coefficient of variation.

#### 4.1.1. Equivalence Between WAP and MIP Data

**Figure 8:**
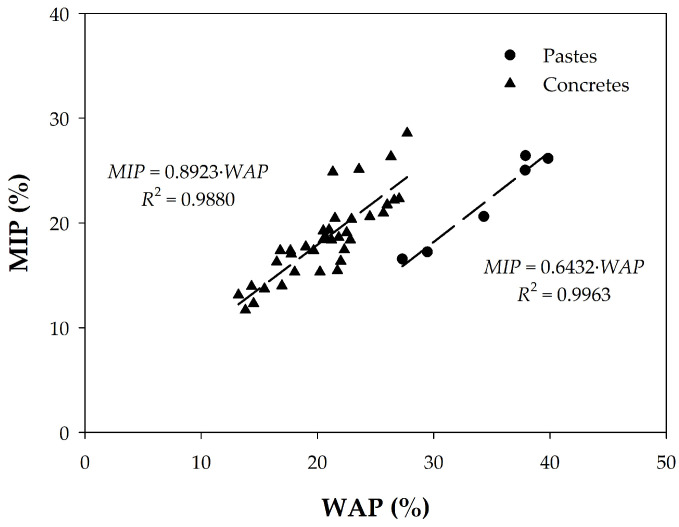
Comparison of MIP porosity values of concretes and pastes with corresponding WAP data.

Furthermore, presenting the data in another comparative way as in Figure 8, we find a very good linear correlation between the two porosities with the exception of concretes H15, H21, H22, H30, and H36, where the porosity value due to mercury intrusion is higher than that accessible to water. This indicates once again that the effect of the aggregate is relatively small and that, therefore, an equivalence can be established between the two types of measurement.

**Figure 9:**
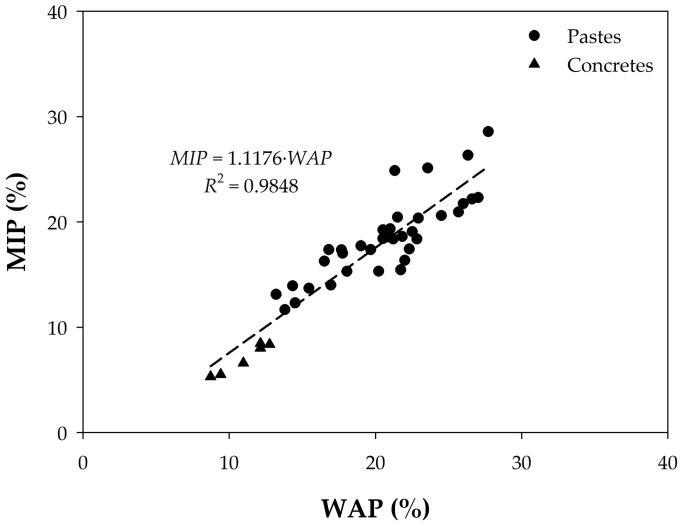
Comparison of total porosity of all samples after adjusting those of the pastes as described in the body of the text.

To find this equivalence, the data of the pastes and concretes were analyzed together. For this purpose, it can be considered that, in a mass of concrete, the approximate average proportions of its components are between 30 and 40% of cement paste, and the aggregates represent between 60 and 70% of the total concrete. Thus, by adjusting, for example, the amount of paste to 32%, the porosities of the cement pastes and the concretes can be represented together, as shown in Figure 9. In this graph, if all the points are considered, a linear relationship with an *R*^2^ = 0.985 is obtained as follows:

(3) MIP=1.18×WAP

However, considering only concrete samples, we obtain a worse regression coefficient (*R*^2^ = 0.7093 in Figure 8), with the expression

(4) MIP=0.8923×WAP

These expressions contribute to the main objective of this work, which is to investigate whether total porosity measured with water under vacuum can replace MIP as a characterization method for concrete quality control. MIP presents lower values than WAP in most cases, probably because water, as a fluid, is also able to penetrate air and larger pores, or because the surface tension in MIP does not adapt to all pore shapes and sizes, as explained below.

#### 4.1.2. Corrections to WAP Values to Get Closer to MIP Data

A possible cause of the differences between the porosity values determined by each method could come from the fact that when applying the Washburn–Laplace expression (Equation (1)) in MIP, the pore shape is not taken into account and, therefore, the variation in surface tension as a function of the pore size [54]. In fact, this equation assumes that *γ* remains constant throughout the test, which might be incorrect, as mentioned. When the radius of curvature of the mercury meniscus (*r*) decreases from infinity, it can be assumed that its surface tension may decrease. Given this consideration, the following generalized Washburn–Laplace expression was proposed [54]:

(5) $$d=\frac{-\phi \cdot \gamma_{\infty }\cdot \cos\theta }{p}+4\cdot b\cdot \cos\theta$$

where *ϕ* is the pore shape factor, which varies from 2 to 4 [54]. With this modification, corrections of up to 30% are obtained for pressures below 100 MPa.

**Figure 10:**
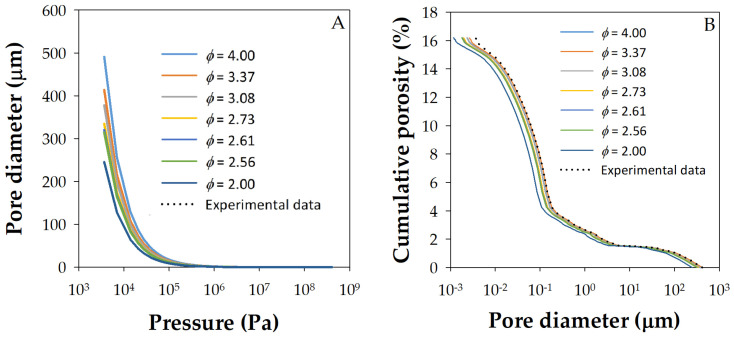
(**A**) Effect of pore factor on pore diameter, and (**B**) effect of the pore factor on the cumulative porosity as a function of pore diameter for sample H18. MIP data with *γ* = 0.481 N/m, *θ* = 141°, and *ϕ* varying between 2 and 4, as indicated in the figure.

Figure 10A shows the effect that different sphericities have on the pore diameter. It is observed that, as the shape factor decreases, the value of *d* (pore diameter) also decreases, reaching values almost 50% lower than those of a pore with a circular cross-section. This implies a considerable variation in the surface tension of mercury throughout the experiment, given the tortuous nature of cement and concrete pores. It also influences the pore size distribution as a function of the volume of mercury introduced for a given operating pressure. Therefore, for low values of the shape factor, *ϕ*, higher pressures must be used to obtain the same cumulative porosity of the sample, as shown in Figure 10B. From these data, it was obtained that the shape factor that best fits the experimental results is *ϕ* = 3.37 for the tested samples, i.e., considering pores of elliptical geometry 1.5:1. And for small diameters, of the order of 10 nm and less, the best fits are obtained for the shape factor 4.

Typically, it is possible to achieve an adjustment of about 10–15% of the observed difference between the two porosity measurements using this correction procedure. However, for practical reasons, the increased processing complexity might not compensate for the increased accuracy achieved, so this additional recalculation on the MIP data is not recommended as a general requirement for routine quality control.

### 4.2. Additional Calculations from MIP and WAP Data

Porosity data can also be analyzed in relation to the characteristics of concrete such as the water/cement ratio and mechanical strength, to derive information on the tortuosity of the pores in the samples, and to obtain a frost durability factor.

#### 4.2.1. Tortuosity Factor

**Figure 11:**
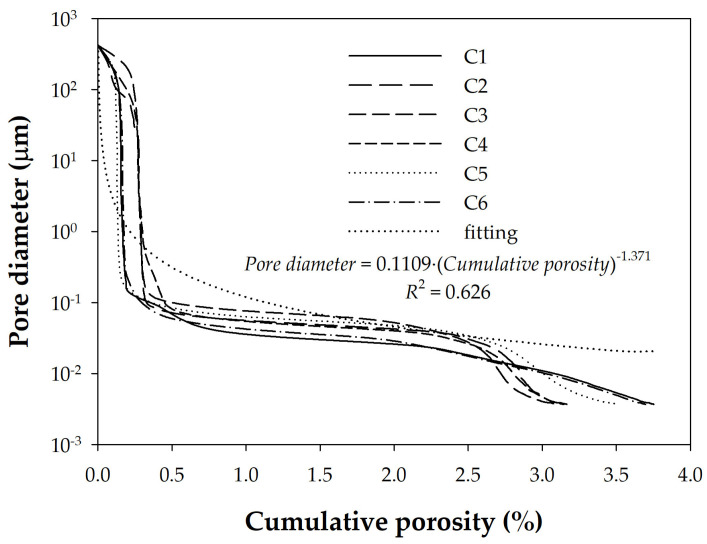
Fitting a potential function to the cumulative porosity distribution curve of pastes obtained with MIP.

Taking advantage of the results obtained with the large set of different concretes, it was investigated whether they also exhibited different tortuosity in their pores using the approach proposed in [42]. Therefore, related to pore shape, tortuosity is a relatively debated concept, with no consensus having been reached on how to quantify it for cement-based materials. In [42], instead of defining tortuosity, a quantitative criterion was adopted to characterize a “tortuosity factor”, which is defined as the exponent on the porosity distribution curve with the pore size, provided that porosity is plotted on the *X*-axis and pore size on the *Y*-axis (cf. Figure 11). In other words, the tortuosity factor can be derived from the shape of the MIP curve by fitting the following equation:

(6) $$\phi_{th}=\phi_{0}\cdot \varepsilon^{-\tau }$$

where *ϕ_th_* is the pore diameter at any porosity, *ϕ*_0_ is the smallest diameter that can be measured in the MIP equipment used (3.6 × 10^−9^ m in present case), *ε* is the porosity, and *τ* is the tortuosity factor.

Figure 11 shows a pore size distribution, but represented in such a way that fitting Equation 6 allows the tortuosity factor *τ* to be deduced. Thus, the volumetric fraction (cumulative porosity) has been plotted on the *X*-axis and the pore diameter on the *Y*-axis. Tortuosity factor values close to 2 were found for typical pores in Portland cement. Therefore, lower values imply slightly tortuous pores, while concretes with values *τ* > 2 indicate notable tortuosity, usually generated by the presence of mineral additions.

**Figure 12:**
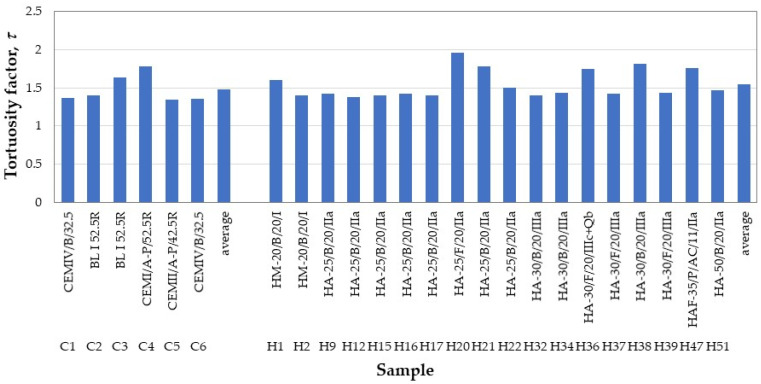
Tortuosity factors for paste and concrete samples.

**Figure 13:**
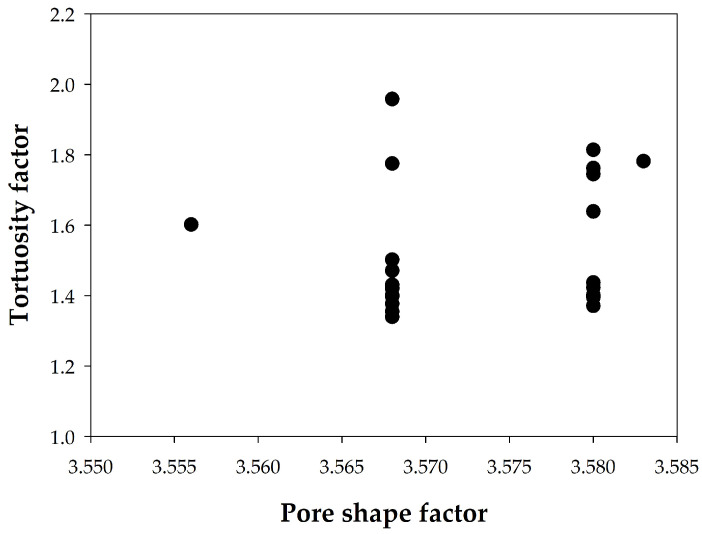
Relationship between tortuosity and pore shape factors.

The values of the tortuosity factor obtained for 25 of the samples are shown in Figure 12. All values, both for pastes and concretes, are lower than 2, with those for pastes being slightly lower than those for concretes. This finding is unusual, as cements contain mineral additions and should have presented higher tortuosity factors. A similar comment can be made for the concrete samples due to their low values despite the presence of mineral additions in all cases, which could be attributed to the use of basaltic aggregates, whose porosity is mainly due to larger pores, between 10 and 100 µm. Following the proposal by Saffiudin and Hearn [47], the tortuosity factor was plotted as a function of the shape factor *ϕ* in Figure 13, but a specific relationship between them could not be established, since the tortuosity varies greatly for the same shape factor. Therefore, it is necessary to further characterize the influence of the geometry and surface irregularities of concrete pores on the total porosity values.

#### 4.2.2. Relationship Between Porosity and Water/Cement Ratio

**Figure 14:**
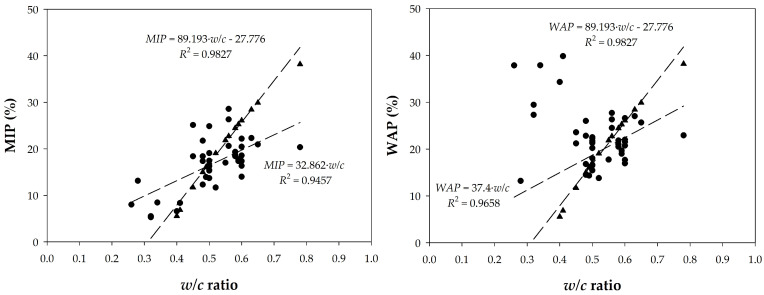
Trends in porosity values with *w/c* ratio of pastes and concretes (●) compared to the theoretical trend of Powers (▲).

The relationships between the porosity and water/cement (*w*/*c*) ratio were then analyzed. Figure 14 shows the representations of the MIP and WAP measurements as a function of the *w*/*c* ratios of the pastes (corrected by their proportion in the concrete) and concretes, finding a correlation with a high regression coefficient. In the same graph, the values of the porosity calculated according to Powers [4,54,55,56,57] are presented. These were obtained from capillary porosity and considering a degree of hydration, *α*, of 100%, using Equation (7).

(7) ε(%volume)=wc−0.36⋅αwc+0.32⋅100

Although this equation is strictly applicable only to pastes prepared with pure Portland cement, which is not the case, it has been verified that the data do not differ significantly from those obtained up to a *w/c* ratio = 0.7, despite the large dispersion between the experimental values. The slope of the relationship is certainly different between Powers’ data and the measured data, but they have an average value of the same order. The trends are similar in terms of WAP and MIP. It is certain that the trends and slopes would improve if the degree of hydration of each sample was known.

#### 4.2.3. Relationship Between Porosity and Compressive Strength

Table 1 shows the characteristic compressive strength at 28 days of the cement pastes and concretes tested. It is observed that the highest values of compressive strength at 28 days are presented by the cement paste samples, with values between 53.70 and 74.10 N/mm^2^. The difference observed between the different samples is due to the fact that the type of cement varies from 32.5 to 52.5 N/mm^2^. As for the concretes, the compressive strength values at 28 days vary from 12.18 N/mm^2^ to 58.55 N/mm^2^. It is observed that concretes H2, H20, and H21 give compressive strength values lower than those expected according to the delivery specifications. Thus, concrete H2 has a strength value of 12.18 N/mm^2^, much lower than the characteristic compressive strength of concrete of 20 N/mm^2^ according to its identification, while H20 and H21 have values of 24.81 and 20.25 N/mm^2^, lower than the nominal 25 N/mm^2^. Finally, as the characteristic compressive strength of concrete increases, the difference between the porosities obtained by the two methods decreases, maintaining, in the case of concrete, an excellent linear correlation.

**Figure 15:**
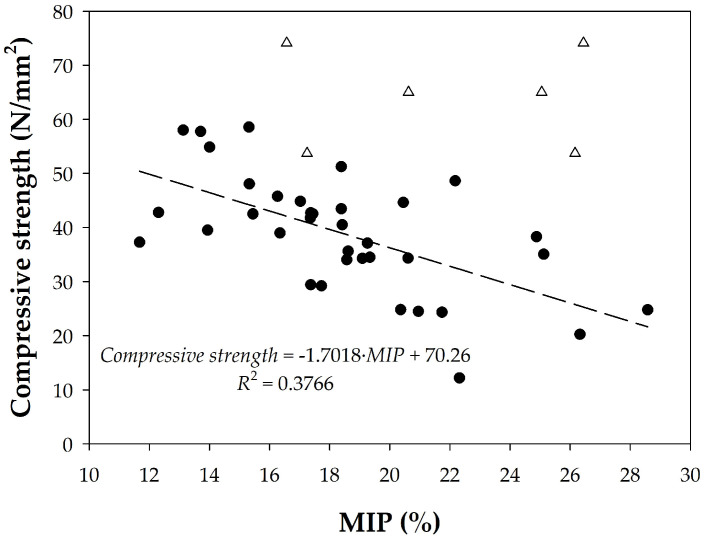
Relationship between compressive strength and MIP porosity for (△) paste and (●) concrete samples.

When the relationship between the observed porosity and the compressive strength at 28 days is taken into account, the results shown in Figure 15 are obtained. It is observed that as the porosity of the sample increases, the compressive strength generally decreases, which is the expected behavior. The cement paste samples are the only ones that do not follow this trend for some of the cases analyzed. The resulting linear function has a low regression coefficient of *R*^2^ = 0.3766 (cf. Figure 15).

**Figure 16:**
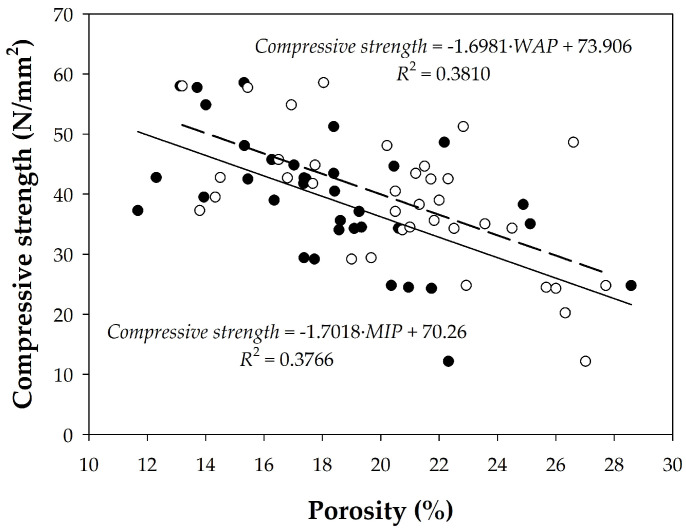
Relationships between compressive strength and porosity values for concrete samples obtained using either (●) MIP or (○) WAP methods.

In Figure 16, only concretes with porosities measured by both techniques were considered. Although there is a trend, the regression coefficients are, once again, quite low. However, analyzing these data by grouping the concretes, we observe a higher *R*^2^ with a linear relationship between the characteristic strength and observed porosity for HM20 and HA25 concretes (cf. Table 3), and they maintain the same trend as that observed for the entire group of samples. However, the second group, consisting of HA30, HA35, and HA50 concretes, shows a weaker correlation between the compressive strength and porosity data, regardless of the porosity determination method. Within this group, if we separate the HA30 concrete, we observe that, in fact, it presents the same trend as the previous group (HM20 and HA25 concretes). The observed dispersion could be due to the fact that HA35 is self-compacting and HA50 contains 12 mm aggregates, achieving minimal difference between porosities regardless of the method used. It should be noted that the concretes used are not laboratory concrete, but rather come from real construction sites.

In conclusion, the generic compressive strength–porosity relationship found by Powers is again generalizable only in trends, but it is not sufficiently precise in individual cases given the many variables involved in a composite material such as concrete, which continuously evolves over time.

#### 4.2.4. Porosity and Frost Resistance

**Figure 17:**
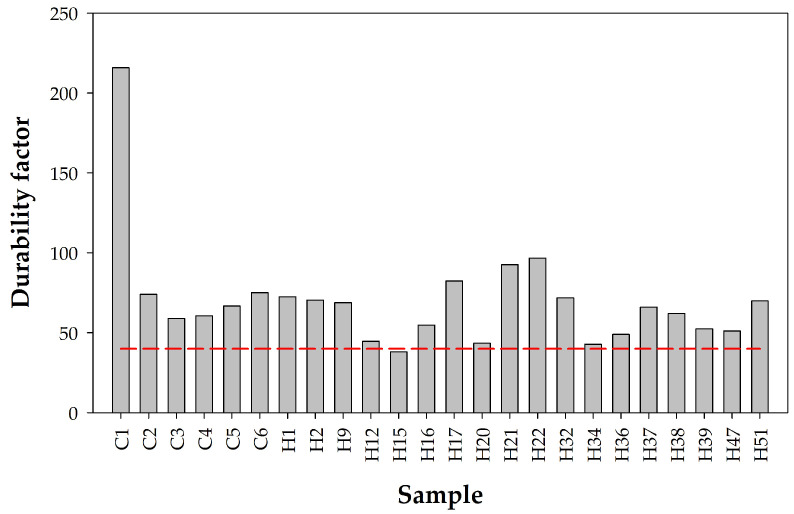
Frost durability factor for all samples under study. The red horizontal line represents the threshold above which the samples meet the requirements. It is observed that all samples are resistant to frost with the sole exception of H15.

Another parameter directly related to the porosity of a cementitious sample is the resistance to severe freeze–thaw cycles, conditions that can occur at high altitudes on the island of Tenerife. This variable is related to the pore structure of the aggregate. Kaneuji et al. [58] developed a method to quantify it, which is summarized in the following equation

(8) $$EDF=\frac{0.579}{V_{4.5}}+6.12\cdot d_{m}+3.04$$

where *EDF* is the durability factor of the aggregate, with a threshold value of 40 (below this value is considered poor), *V*_4.5_ is the total mercury intrusion volume, in cm^3^/g, for a pore diameter of 4.5 nm, and *d_m_* is the average pore size, in µm. Figure 17 shows the freeze–thaw durability factors for a set of tested samples. It is observed that all the analyzed samples, except one (H15), have acceptable durability values.

**Figure 18:**
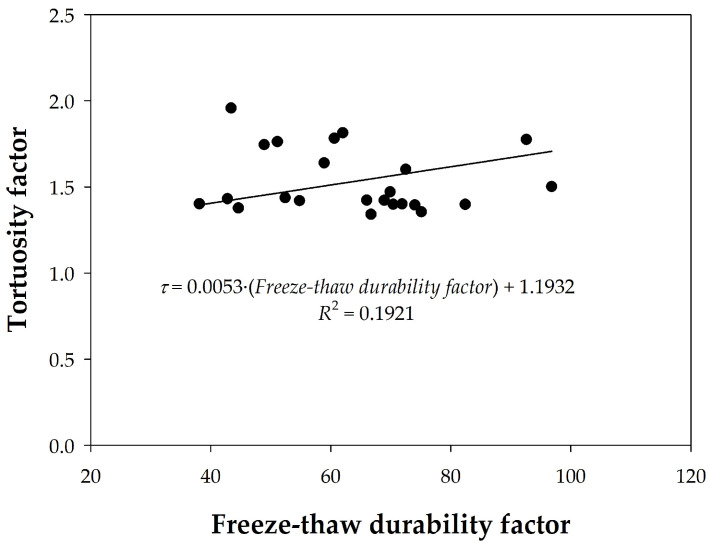
Relationship between tortuosity and frost durability factors.

Finally, in Figure 18, these frost durability factors have been plotted against the tortuosity factors, a parameter also very critical for frost resistance, finding a very low correlation coefficient. However, the durability factor tends to increase as the tortuosity increases, which seems contradictory, since one would expect the durability against frost to decrease with greater tortuosity. Therefore, it is necessary to continue searching for parameters that may complement the porosity, as well as to control every characteristic or process related to the durability of concrete.

## 5. Conclusions

In this study, the applicability of dynamic water vapor sorption (DWVS) was evaluated as a suitable technique for characterizing cement-based materials instead of the commonly used mercury intrusion porosimetry (MIP) method to avoid the use of mercury, which is a hazardous substance subject to banning regulations, while the former is much simpler to implement. Porosity was chosen because it is one of the characteristics of concrete related to both strength and durability. Although MIP data provide a more complete description of the fundamental characteristics of concrete samples, while DWSV only accessed total porosity, the use of MIP in control analyses does not necessarily require further characterization. Therefore, porosity by water intrusion under vacuum (referred to as WAP) was selected and the equivalence between the two was investigated.

A wide range of cementitious materials, including mortar, paste, and aggregate obtained from concrete, were considered to assess the differences between the MIP and WAP data. In other words, the equivalence between the methods and MIP and DWSV data was studied. Cylindrical specimens manufactured on site in small and large concrete mixers were used for a study related to the search for complementary characteristics of concrete to strength, in order to verify the consistency of the quality of concrete production. This work was undertaken due to the limited literature available on the equivalence of porosity data obtained by mercury intrusion porosimetry and with water under vacuum conditions. The main conclusions of this work are as follows:

1. The porosity of the pastes was generally, but not entirely, greater than that of the concretes.
2. Similarly, the MIP results were generally smaller than the WAP results, but not in all cases.
3. The following linear relationships with high *R*^2^ were found:
  MIP = 1.18 × WAP (with *R*^2^ = 0.9848) for all concretes, as well as pastes after application of a relevant adjustment procedure (i.e., fitted);
  MIP = 0.8923 × WAP for concretes alone (with *R*^2^ = 0.7093);
  Which makes WAP a suitable alternative to MIP for quality control.
4. Regarding the additional information that can be extracted from the porosity measurements, in this work, the following was found:
  - Tortuosity factors calculated by fitting the MIP pore size distribution with a power law were less than a value of 2, typical of Portland cement, in both pastes and concretes.
  - No relationship was found between these tortuosity factors and the pore shape factors obtained by varying the pore geometry factor and the surface tension in MIP measurements.
  - Calculation of porosity by Powers formula for capillary porosity presented a different trend with the water/binder ratio than the values found by MIP or WAP, although the values for typical *w/c* ratios of concrete were quite close.
  - The relationship between porosity and strength suggested by Powers was found, but with low *R*^2^ values, although improved when the concretes were grouped into several “families”.
  - The frost durability factor calculated following the literature had a coherent trend with the tortuosity factors calculated in the present work.
5. Porosity is the characteristic of concrete that is often related to its durability, but its determination faces several difficulties. The first of these is the selection of the measurement technique, followed by the establishment of significant relationships between the porosity and relevant characteristics of concrete. Much remains to be performed to achieve a more in-depth understanding than is currently the case.

## Figures and Tables

**Table 1:** Sample types and main characteristics, as well as the corresponding compressive strength.

Sample Code	Type	*w*/*c* Ratio	Cement Content (kg/m^3^)	Compressive Strength at 28 Days (N/mm^2^)
Cement pastes				
C1	CEMIV/B/32.5	0.34		74.10
C2	BL I 52.5R	0.32		53.70
C3	BL I 52.5R	0.40		65.00
C4	CEMI/A-P/52.5R	0.32		74.10
C5	CEMII/A-P/42.5R	0.41		53.70
C6	CEMIV/B/32.5	0.26		65.00
Concretes				
H1	HM-20/B/20/I	0.65	275	24.49
H2	HM-20/B/20/I	0.63	250	12.18
H3	HA-25/B/20/IIa	0.59	344	29.40
H4	HA-25/B/20/IIa	0.59	344	29.20
H5	HA-25/B/20/IIa	0.52	350	37.27
H6	HA-25/B/20/IIa	0.58	340	37.12
H7	HA-25/B/20/IIa	0.60	340	44.64
H8	HA-25/B/20/IIa	0.58	340	35.61
H9	HA-25/B/20/IIa	0.60	350	54.86
H10	HA-25/F/12/IIa	0.56	324	34.33
H11	HA-25/B/20/IIa	0.55	355	44.84
H12	HA-25/B/20/IIa	0.60	390	34.05
H13	HA-25/F/12/IIa	0.58	330	40.50
H14	HA-25/F/12/IIa	0.58	330	34.50
H15	HA-25/B/20/IIa	0.50	325	38.28
H16	HA-25/B/20/IIa	0.50	300	42.54
H17	HA-25/B/20/IIa	0.5	300	42.50
H18	HA-25/F/20/IIIa	0.5	370	48.07
H20	HA-25/F/20/IIa	0.78	300	24.81
H21	HA-25/B/20/IIa	0.56	300	20.25
H22	HA-25/B/20/IIa	0.56	240	24.79
H29	HA-30/B/20/IIIa	0.48	390	42.77
H30	HA-30/B/20/IIIa	0.48	395	42.70
H31	HA-30/B/20/IIIa	0.49	385	39.50
H32	HA-30/B/20/IIIa	0.50	370	57.75
H33	HA-30/B/20/IIIa	0.50	360	45.75
H34	HA-30/B/20/IIIa	0.50	350	58.55
H35	HA-30/F/20/IIIc + Qb	0.45	370	43.46
H36	HA-30/F/20/IIIc + Qb	0.45	360	35.06
H37	HA-30/F/20/IIIa	0.50	360	34.28
H38	HA-30/B/20/IIIa	0.60	365	38.99
H39	HA-30/F/20/IIIa	0.48	300	24.32
H46	HAF-35/P/AC/11/IIa	0.60	380	41.77
H47	HAF-35/P/AC/11/IIa	0.60	380	48.63
H50	HA-50/F/12/IIa	0.28	500	57.99
H51	HA-50/B/20/IIa	0.48	380	51.24

**Table 2:** Composition of concrete samples (% by weight).

	Cement	Basaltic Gravel	Basaltic Sand	African Sand	Water	Total Mass
Min., %	10.94	36.23	20.71	9.26	6.58	2135
Max., %	15.47	44.31	31.71	19.88	7.43	2263
Med., %	13.15	41.42	24.57	13.45	6.90	2193.2

**Table 3:** Dry density, porosity values, and tortuosity factors obtained from the samples.

Sample Code	Dry Density at 70 °C (g/cm^2^)	Water-Accessible Porosity (%)	Mercury Intrusion Porosity (%)	Tortuosity Factor |*τ*|
Aggregates				
A1	2.66	8.00	18.80	
A-635 A1	2.80		14.4	
A-635 A2	2.24		36.2	
A-635 A3	2.57		16.9	
A-365 B1	2.35		1.3	
A-365 B2	2.72		12.2	
A-365 B3	2.85		6.27	
Cement pastes				
C1	1.57	37.89	26.44	1.371
C2	1.75	29.45	17.25	1.395
C3	1.59	34.31	20.62	1.639
C4	1.71	27.30	16.57	1.782
C5	1.46	39.83	26.16	1.34
C6	1.58	37.86	25.05	1.355
Concretes				
H1	2.14	25.67	20.95	1.602
H2	1.96	27.02	22.32	1.399
H3	2.19	19.67	17.37	
H4	2.14	19.00	17.73	
H5	2.20	13.80	11.68	
H6	2.20	20.50	19.26	
H7	2.18	21.50	20.45	
H8	2.20	21.83	18.62	
H9	2.25	16.94	14.01	1.422
H10	2.07	24.50	20.61	
H11	2.22	17.75	17.03	
H12	2.18	20.74	18.57	1.377
H13	2.15	20.50	18.42	
H14	2.24	21.00	19.34	
H15	2.23	21.32	24.88	1.402
H16	2.17	22.31	17.44	1.42
H17	2.23	21.72	15.45	1.398
H18	2.24	20.21	15.33	
H20	2.15	22.93	20.36	1.958
H21	2.05	26.32	26.33	1.775
H22	1.98	27.71	28.58	1.502
H29	2.28	14.50	12.31	
H30	2.17	16.80	17.37	
H31	2.19	14.33	13.94	
H32	2.32	15.44	13.71	1.401
H33	2.27	16.50	16.27	
H34	2.27	18.04	15.32	1.431
H35	2.19	21.20	18.39	
H36	2.14	23.57	25.12	1.745
H37	2.14	22.51	19.09	1.423
H38	2.22	22.00	16.35	1.814
H39	1.97	26.00	21.74	1.437
H46	2.19	17.67	17.36	
H47	1.87	26.60	22.18	1.762
H50	2.31	13.20	13.13	
H51	2.10	22.83	18.39	1.471

## Data Availability

The original contributions presented in this study are included in the article. Further inquiries can be directed to the corresponding authors.
